# Clinical significance of hyperuricaemia in biopsy-proven diabetic kidney disease ━ a single-centre retrospective study

**DOI:** 10.3389/fendo.2025.1481977

**Published:** 2025-01-24

**Authors:** Jin Yu, Xiao Tu, Kunyue Xu, Xuanli Tang, Yufan Wu, Xue Jiang

**Affiliations:** ^1^ Department of Nephrology, Hangzhou Traditional Chinese Medicine (TCM) Hospital Affiliated to Zhejiang Chinese Medical University, Hangzhou, China; ^2^ Key Laboratory of Precise Prevention and Treatment of Rheumatism Syndrome of Renal Wind Disease, Hangzhou, China

**Keywords:** diabetic kidney disease, hyperuricaemia, prognosis, nomogram model, IFTA

## Abstract

**Aims:**

Hyperuricaemia is associated with the development of Diabetic kidney disease (DKD). However, the mechanism of hyperuricaemia causing the progression of DKD remain unclear.

**Methods:**

This is a single-centre retrospective study. 155 biopsy-proven DKD patients were grouped into hyperuricaemia and non-hyperuricaemia groups. Kaplan-Meier analysis and landmark curves were performed to explore predictors of end-stage renal disease (ESRD), Cox regression analysis was used to screen for factors, a nomogram was constructed to predict the renal prognosis of DKD.

**Results:**

Patients in hyperuricaemia group had higher serum creatinine (Scr), degree of mesangial expansion and IFTA score and lower GFR, haemoglobin. SUA level was positively correlated with IFTA scores. The Kaplan-Meier curve and landmark analysis revealed worse survival in hyperuricaemia group, especially after 12 months. 11 variables, including age, sex, haemoglobin, Scr, SUA, and pathological score were collected to make a nomogram model. In the testing and training sets, the AUCs at 1, 3, and 5 years were 0.888, 0.939, and 0.886 and 0.947, 0.867, and 0.905, respectively.

**Conclusion:**

The clinicopathologic manifestation of DKD patients with hyperuricaemia was much more severe, and hyperuricaemia predicted a worse renal prognosis. A new renal prognosis prediction model including SUA was constructed for DKD with higher accuracy.

## Introduction

Diabetic kidney disease (DKD) occurs in 20–40% of all diabetic patients and is the primary cause of chronic kidney disease (CKD) (1, 2). The percentage of incident end-stage renal disease (ESRD) patients, due to diabetes, increased from 22.1% to 31.3%, and DKD became the main cause of CKD (3). The main pathological manifestations of DKD include thickening of the glomerular basement membrane (GBM), mesangial proliferation, endothelial alteration, and podocyte injury (4). Many factors contribute to the development of DKD.

Hyperuricaemia (HUA) is common among patients with CKD and increases in severity with the decline in excretory kidney function. Whether elevated uric acid levels contribute to the progression of DKD has remained a subject of debate for years. Numerous studies have focused on the effect of serum uric acid (SUA) levels on the progression to diabetic nephropathy in patients with diabetes. Zoppini et al. (5) followed 1449 type 2 diabetes mellitus (T2DM) patients for 5 years, and the highest quartile of initial SUA levels was associated with a significantly higher cumulative incidence of CKD III or an incidence of CKD III that was greater than those in lower quartiles. A total of 2367 patients had T2D follow-up for 4.6 years. The results showed that SUA>6.3 mg/dL was an independent risk factor associated with progression in CKD stage. The progression group had the highest baseline SUA level (6), while the research on allopurinol lowering uric acid carried out by Doria et al. showed that SUA reduction had no beneficial effect on the rate of GFR decline or other kidney outcomes in patients with diabetes (7). Therefore, we need further to explore the real cause of kidney damage caused by SUA, and renal pathology is the most direct means to reflect kidney injury, while the correlation between SUA level and pathological changes in DKD has not been studied. This study focused on patients diagnosed with DKD by renal biopsy and further explored the correlation between SUA level and the clinical, pathological and prognosis of DKD.

## Subjects, materials and methods

### Study population

In this retrospective study, 155 DKD patients with T2DM were selected. From February 2009 to March 2021.

All patients were over 18 years old and met the criteria for the diagnosis of T2DM proposed by the American Diabetes Association (8). The pathological diagnosis of DKD was confirmed by renal pathologists according to the criteria proposed by the Renal Pathology Society in 2010 (9).

The exclusion criteria were as follows: (1) type 1 diabetes mellitus or other special types of diabetes; (2) Cushing’s syndrome, hyperthyroidism, or other endocrine metabolic diseases; (3) serious diseases, such as acute cardiovascular and cerebrovascular diseases or severe infections; (4) serious complications, such as diabetic ketoacidosis and hyperosmolar hyperglycaemic syndrome, or other acute metabolic disorders; and (5) pregnancy or malignancy. The study was conducted according to the Declaration of Helsinki and approved by the Institutional Review Board of Hangzhou Hospital of Traditional Chinese Medicine (No. 2023KLL040).

### Data collection

Clinical data were collected at the time of renal biopsy, including sex, age, duration of diabetes, 24-hour proteinuria (24 h-Upro), serum creatinine (Scr), serum uric acid (SUA), haemoglobin A1c (HbA1c), triglyceride, haemoglobin (Hb), white blood cell count (WBC), body mass index (BMI), albumin, total cholesterol concentrations, low-density lipoprotein (LDL), Erythrocyte sedimentation rate(ESR), Hypersensitive C-reactive protein(hs-CRP), platelet, triglyceride, serum total calcium, blood phosphate, and serum C3. The estimated glomerular filtration rate (eGFR) was calculated by the Chronic Kidney Disease Epidemiology Collaboration equation (EPI). Patients were considered to have hyperuricaemia when their initial SUA levels were ≥420 μmol/l for men and ≥360 μmol/l for women (using the criteria of the Czech Rheumatologic Society (31)). We also collected the number of patients who received urico-lowering therapy.

### Histological parameters

After renal biopsy, all renal samples were evaluated and graded by two pathologists. Light microscopy, direct immunofluorescence, and electron microscopy were routinely performed for each renal biopsy specimen. Glomerular lesions were classified into classes I, II, III, or IV (8) based on glomerular basement membrane thickening, degree of mesangial expansion (1, <25%; 2, 25–50%; 3, >50%), presence of nodular sclerosis (Kimmelstiel-Wilson lesions), and advanced diabetic glomerulosclerosis. On the basis of the affected proportion of the tubulointerstitial compartment, interstitial fibrosis and tubular atrophy (IFTA) were semiquantitatively scored as 0, 1, 2, or 3 (0, absent; 1, <25%; 2, 25–50%; 3, >50%). Interstitial inflammation was graded according to the infiltrated area (0, absent; 1, infiltration only in areas related to IFTA; 2, infiltration in areas without IFTA). Vascular changes were evaluated based on arteriolar hyalinosis and large vessel arteriosclerosis. arteriolar hyalinosis (0, absent; 1, at least one arteriolar hyalinosis is present), large vessel arteriosclerosis (0, absent; 1, at least one large vessel arteriosclerosis present)). Electron microscopy was performed to exclude some renal morphologic lesions, such as podocyte fusion, and podocyte fusion was scored as 0 or 1 (0, <50%; 2, ≥50%).

### Follow-up and outcome

Subjects were followed-up for a median of 31 [IQR 16–41] months until the primary end point or the end of the research (Mar. 2023). The primary end point was progression to ESRD, which was defined as a requirement of permanent renal replacement therapies for >3 months. If the patient was shed or lost to follow-up, all the data of the study object could not be observed after the follow-up time point, at this time, we used the single filling method to fill in the missing data.

### Analysis

Statistical analyses were performed using Statistical Package for Social Science Software version 26.0. The results are expressed as the mean ± standard deviation (SD) for continuous normally distributed data or median and interquartile range (IQR) for continuous nonnormally distributed data. Categorical variables are expressed as percentages and were compared with Pearson’s chi-square test. Correlations of clinicopathological data were performed by linear correlation analysis. Multivariate Cox regression was performed to explore potential predictors of renal outcomes. The results are expressed as hazard ratios (HRs) and 95% confidence intervals (95% CIs), and p<0.05 was considered statistically significant. Meanwhile, Risks in different subgroups were analyzed based on the incorporating statistical tests for interaction terms. Kaplan-Meier survival curves and landmark curves were plotted using R software (Version 4.0.5, https://CRAN.R-project.org/mirrors.html).

### Construction and validation of a risk prediction model

A total of 155 patients were randomly divided into a training set (70%) and a testing set (30%). Patients in the training set were used to develop the nomogram model, whereas patients in the testing set were used to validate the resulting nomogram.

Based on the multiple Cox proportional hazard model, the independent risk factors obtained based on the multivariate regression method were used to construct a nomogram model with the “rms” package of R software (version 3.6.1). We used the Schoenfeld test for the proportional hazards assumption. Finally, we used the time-dependent ROC (tdROC) curve to assess the predictive accuracy and reliability of the final model, and one, three, and five years of follow-up were calculated. The AUC values ranged from 0.5 to 1.0, with low accuracy (<0.5), moderate accuracy (0.5–0.7), and high accuracy (>0.7).

## Results

### Clinical data and baseline data analysis

**Figure 1:**
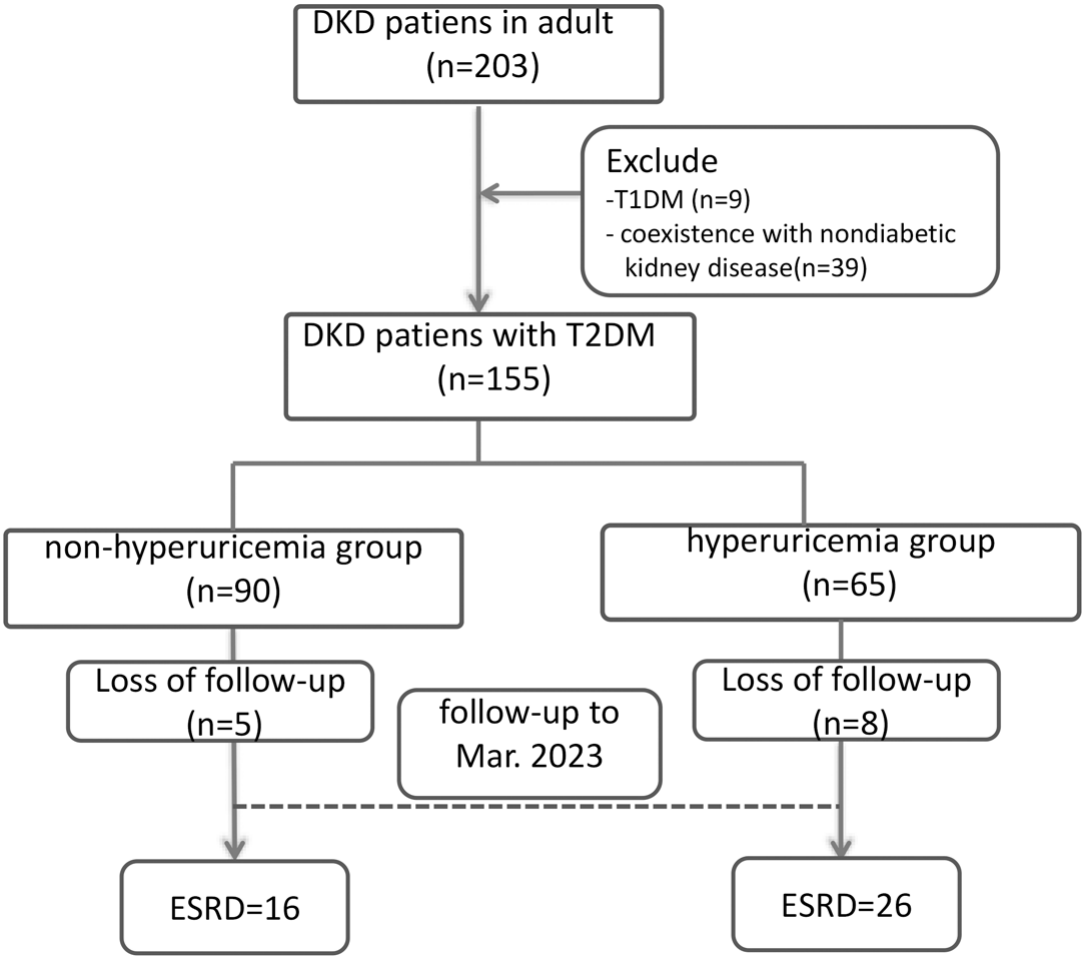
Patient flow chart. DKD, Diabetic kidney disease; T2DM, type 2 diabetes mellitus; T1DM, type 1 diabetes mellitus; ESRD, end-stage renal disease.

A total of 155 of 203 DKD patients were included in the final analysis. The median age of the 155 cases was 50 years (IQR, 44-60), 116 were male and 39 were female. The median duration of DM was 92 (30, 126) months. The median level of urinary protein was 3.5 (1.0, 4.7) g/24 h. The serum albumin level was 33 (30.4, 38.7) g/L. The median serum creatinine was 153.0 (81, 267) μmol/L, and the EPI-eGFR was 66 (41, 90) mL/min/1.73 m^2^. All patients were divided into a hyperuricaemia group (N=65) and a nonhyperuricaemia group (N=90) according to whether they had hyperuricaemia (**Figure 1**).

Comparing patients with and without baseline hyperuricaemia, our study revealed significantly higher serum creatinine, total cholesterol, low-density lipoprotein, and blood phosphate concentrations and lower GFR and haemoglobin levels in hyperuricaemia subjects (p<0.05). More patients received uric acid-lowering therapy in hyperuricaemia group than in nonhyperuricaemia group (**Table 1**).

**Table 1:** Baseline of pathological classification factors of DKD patients with and without hyperuricemia.

	non-hyperuricemia group N=90	hyperuricemia group N=65	P value
age	51.36 ± 10.39	50.82 ± 10.84	0.754
Diabetes duration	7.80 ± 5.74	7.47 ± 6.79	0.749
BMI	25.35 ± 3.00	25.08 ± 4.01	0.633
24h-Upro	3.10 ± 2.89	4.36 ± 3.80	0.085
EPI	75.44 ± 36.96	53.16 ± 38.54	0.0001
HbAlc	7.61 ± 1.93	67.04 ± 1.70	0.062
blood leukocyte	6.54 ± 1.95	6.87 ± 2.13	0.317
hemoglobin	120.38 ± 21.56	111.40 ± 22.38	0.013
blood phosphate	209.79 ± 64.05	219.38 ± 66.13	0.365
serum creatinine	122.62 ± 83.18	195.14 ± 184.23	0.001
sUA	328.66 ± 57.92	466.89 ± 70.54	0.0001
ALB	35.13 ± 6.31	33.22 ± 5.69	0.055
triglyceride	2.13 ± 1.90	2.72 ± 2.26	0.083
cholesterol concentrations	4.70 ± 1.43	5.24 ± 1.61	0.032
LDL	2.84 ± 1.05	3.22 ± 1.23	0.041
hs-CRP	1.39 (0.7, 2.78)	1.41 (0.58, 2.61)	0.154
ESR	31.13 ± 25.30	35.91 ± 29.88	0.330
No.of receiving urico-lowering therapy	14 (15.5%)	24 (36,92%)	0.004
CKD 1-3	83 (92.2%)	46 (70.8%)	0.001
CKD 4-5	7 (7.8%)	19 (29.2%)	

DKD, Diabetic kidney disease; BMI, body mass index; EPI, Epidemiology Collaboration equation.

LDL, low density lipoprotein; 24h-Upor, 24-hour proteinuria; sUA, serum uric acid; ALB, albumin.

HbA1c, haemoglobin A1c. *P<0.05,**P<0.01,***P<0.001.

The detailed pathological characteristics of these DKD patients are presented in **Table 2**. Compared to patients without hyperuricaemia, DKD patients with hyperuricaemia had a higher degree of mesangial expansion and a significantly higher grade of IFTA (**Table 2**).

**Table 2:** Baseline of clinical characteristics in 155 DKD patients with and without hyperuricemia.

	non-hyperuricemia group N=90	hyperuricemia group N=65	P value
Glomerular lesions classfication	II 51 (56.7%)	II 29 (44.6%)	0.141
	III 37 (41.1%)	III 37 (56.9%)	
	IV 2 (2.2%)	IV 2 (3.1%)	
degree of mesangial expansion	1 35 (38.9%)	1 19 (29.2%)	0.083
	2 51 (56.7%)	2 37 (56.9%)	
	3 4 (4.4%)	3 13 (8.4%)	
degree of endothelial cell proliferation	0 52 (57.8%)	0 37 (56.9%)	0.915
	1 38 (42.2%)	1 28 (43.1%)	
Mesangial cell proliferation classification	1 71 (79.8%)	1 38 (58.5%)	0.015
	2 13 (14.6%)	2 21 (32.3%)	
	3 5 (5.6%)	3 6 (9.2%)	
Kimmelstiel-Wilson lesions	0 56 (62.2%)	0 40 (61.5%)	0.931
	1 34 (37.8%)	1 25 (38.5%)	
Cellulose exudation	0 62 (68.9%)	0 37 (56.9%)	0.126
	1 28 (31.1%)	1 28 (43.1%)	
Proportion of glomerulosclerosis	0.17 ± 0.15	0.22 ± 0.18	0.112
GBM thickness	750.97 ± 189.93	751.85 ± 232.54	0.983
Interstitial inflammation grade	1 84 (93.3%)	58 (89.2%)	0.363
	2 6 (6.7%)	7 (10.8%)	
IFTA	1 18 (20.0%)	6 (9.2%)	0.031
	2 59 (65.6%)	40 (61.5%)	
	3 13 (14.4%)	19 (29.2%)	
interstitial fibrosis	1 27 (31.8%)	1 14 (25.5%)	0.329
	2 30 (35.3%)	2 16 (29.1%)	
	3 28 (32.9%)	3 25 (45.5%)	
vessel arteriosclerosis	0 37 (41.7%)	0 26 (40.0%)	0.49
	1 49 (54.4%)	1 33 (50.8%)	
	2 4 (4.4%)	2 6 (9.2%)	
arteriolar hyalinosis	0 4 (4.4%)	0 3 (4.6%)	0.535
	1 55 (61.1%)	1 34 (52.3%)	
	2 31 (34.4%)	2 28 (43.1%	
podocyte fusion	1 32 (35.6%)	1 16 (25%)	0.163
	2 58 (64.4%)	2 48 (75.0%)	

IFTA, interstitial fibrosis and tubular atrophy; DKD, Diabetic kidney disease. *P<0.05.

### Correlation analysis of SUA and pathological factors

Linear correlation analysis of SUA and the other pathological findings was performed. The results showed that the SUA level was positively correlated with semiquantitative IFTA scores (r = 0.22, *p* =0.006) and degrees of mesangial expansion (r = 0.*159, p =* 0.048). There was no relationship between uric acid and other indices.

### Relationship between SUA and renal survival

We further analysed the association of baseline SUA level and renal prognosis of these patients. Of the initial 155 patients in the study, 8 in the hyperuricaemia group and 5 in the non-hyperuricaemia group were lost to follow-up, leaving 142 patients included in the survival analysis. During the follow-up period, 16 (18.8%) cases in the non-hyperuricaemia group and 26 (44.8%) cases in the hyperuricaemia group met ESRD.

**Figure 2:**
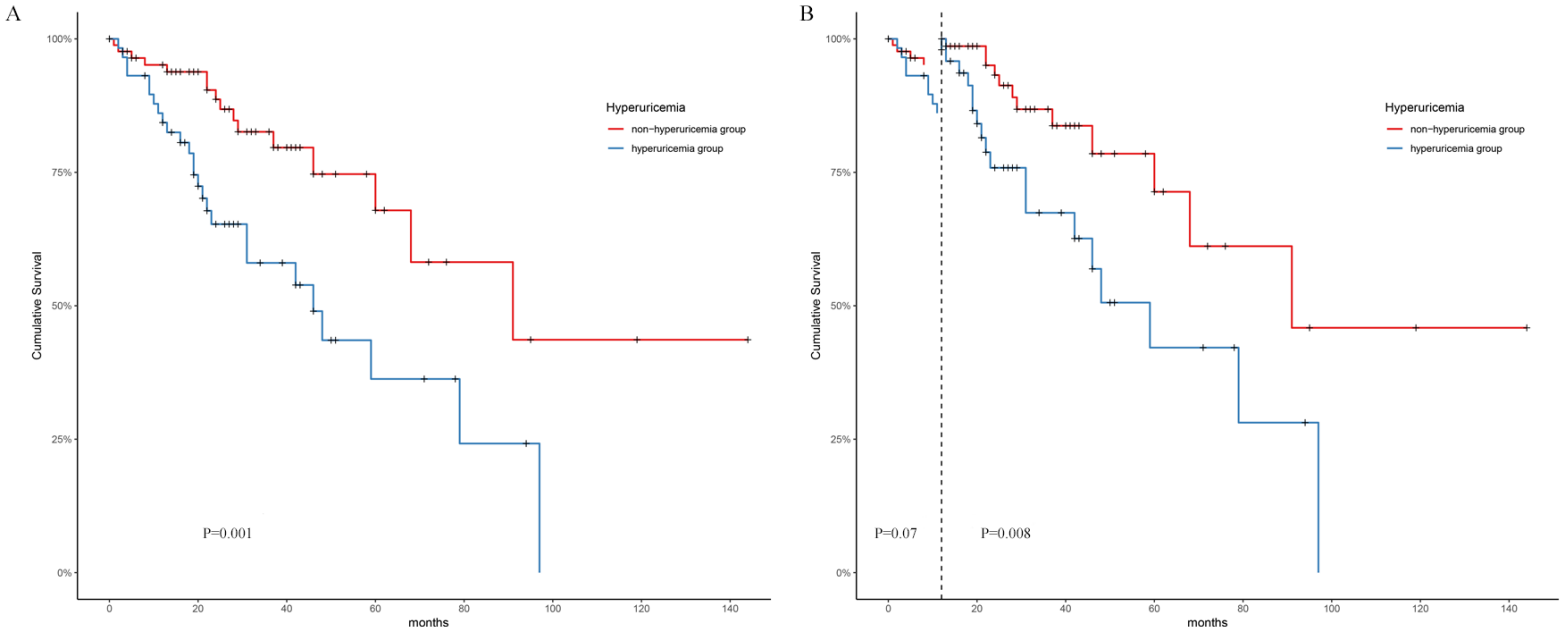
Survival analysis on two groups. **(A)** Kaplan-Meier survival analysis. **(B)**: Landmark analysis.

Kaplan-Meier analysis revealed a significant difference in the cumulative renal survival among the two groups (log-rank χ2 = 10.37, p=0.001). As shown in **Figure 2A**, patients with normal SUA levels had a significantly longer estimated median time to DKD progression (96 months) than the nonhyperuricaemia group, where the estimated median time was 46 months. The 1-, 3-, and 5-year survival rates of the nonhyperuricaemia group were 95%, 88.7%, and 79.6%, respectively. For the hyperuricaemia group, the respective survival rates were 82.5%, 65.3%, and 24.2%. Landmark analysis revealed that patients in the hyperuricaemia group had worse survival than those in the nonhyperuricaemia group after the first follow-up at 12 months (p=0.003), as shown in **Figure 2B**.

### Prognostic factors leading to ESRD in DKD patients

Univariate Cox regression revealed that twelve variables were closely associated with ESRD. These variables included five clinical indices (24 h Upro, Hb, Scr, SUA) and six pathological indices (degree of mesangial expansion, presence of nodular sclerosis, IFTA, interstitial inflammation grade, vessel arteriosclerosis, and podocyte fusion) (**Table 3**).

**Table 3:** Univariate analysis of risk factors associated to ESRD in 155 DKD patients.

Risk factors	Crude HR (95% CI)	p-Value
Clinical index		
24h-Upro	1.20 (1.12-1.28)	<0.001***
Hemoglobin	0.96 (0.94-0.97)	<0.001***
Scr	1.00 (1.00-1.00)	<0.001***
sUA	1.01 (1.00-1.01)	0.002**
ALB	0.93 (0.89-0.97)	0.001***
P	4.62 (1.80-11.84)	<0.001***
Pathological index		
degree of mesangial expansion	2.42 (1.53-3.86)	<0.001***
presence of nodular sclerosis	2.33 (1.25-4.35)	0.008**
IFTA	4.37 (2.45-7.80)	<0.001***
Interstitial inflammation grade	2.28 (1.38-3.77)	0.001***
large vessel arteriosclerosis	2.01 (1.13-3.57)	0.017*
Podocyte fusion	3.22 (1.41-7.37)	0.006**

24h-Upor:24-hour proteinuria;SUA: serum uric acid; ALB: albumin; IFTA:interstitial fibrosis and tubular atrophy; Scr: serum creatinine; HR, hazard ratio; CI, confidence interval. *P<0.05,**P<0.01,***P<0.001.

To investigate the association between possible risk factors and patient kidney survival, a multivariate Cox regression model was conducted. Each potentially modifiable risk factor was adjusted for plausible predetermined confounders (age, sex, BMI, clinical factors: 24 h Upro, haemoglobin, SUA, Scr, pathological classification factors: degree of glomerular lesions, presence of nodular sclerosis, IFTA, interstitial inflammation grade, vessel arteriosclerosis and arteriolar hyalinosis). The results showed that SUA was independently associated with kidney mortality (HR=1.00 (1.00-1.01), p=0.05). When the Scr level was adjusted, SUA had no association with the renal survival rate, as shown in **Table 4**.

**Table 4:** Risk factors associated with ESRD in Cox regression models.

Risk factors	Crude HR (95% CI)	p-Value	Adjusted model^1^ HR (95% CI)	p-Value	Adjusted model^2^ HR (95% CI)	p-Value	Adjusted model^3^ HR (95% CI)	p-Value
Age	0.97 (0.94-1.00)	0.061	0.97 (0.93-1.00)	0.05*	0.95 (0.92-0.98)	0.005	0.95 (0.91-0.98)	0.003**
gender	0.66 (0.33-1.30)	0.228	1.57(0.69-3.62)	0.234	1.72 (0.71-4.19)	0.234	1.70 (0.70-4.12)	0.24
BMI	0.99 (0.91-1.08)	0.873	1.02 (0.92-1.13)	0.73	1.05 (0.96-1.16)	0.303	1.03 (0.93-1.14)	0.59
Hemoglobin			0.96 (0.94-0.98)	<0.001***	0.95 (0.92-0.97)	<0.001	0.95 (0.93-0.97)	<0.001***
24h-Upro			1.15 (1.05-1.26)	0.003**	1.13 (1.04-1.24)	0.007	1.14 (1.04-1.25)	0.004**
degree of mesangial expansion					0.64 (0.31-1.34)	0.641	0.82 (0.37-1.80)	0.621
presence of nodular sclerosis					1.150.50-2.64)	0.739	1.22 (0.53-2.80)	0.637
IFTA					3.24 (1.52-6.86)	0.002	3.13 (1.47-6.69)	0.003**
Interstitial inflammation grade					1.44 (0.45-4.58)	0.534	1.47 (0.45-4.79)	0.521
arteriolar hyalinosis					1.08 (0.46-2.55)	0.865	0.98 (0.42-2.26)	0.953
large vessel arteriosclerosis					2.16 (1.11-4.21)	0.024	1.98 (1.03-3.81)	0.04*
Scr							1.00 (1.00-1.00)	0.065
SUA	1.00 (1.00-1.00)	0.002	1.00 (1.00-1.01)	0.031	1.00 (1.00-1.01)	0.053	1.00 (0.99-1.01)	0.179

Model 1 is adjusted for age, gender, BMI, and clinical factors (24h-Upro, Hb).

Model 2 is adjusted for age, gender, BMI, and clinical factors and pathological classification factors (degree of mesangial expansion, presence of nodular sclerosis, IFTA, interstitial inflammation grade, vessel arteriosclerosis and arteriolar hyalinosis).

Model 3 adjusted for age, gender, BMI, and clinical factors and pathological classification factors and finally Scr.

BM, body mass index; 24h-Upor, 24-hour proteinuria; SUA, serum uric acid; IFTA, interstitial fibrosis and tubular atrophy; Scr, serum creatinine; ESRD, end-stage renal disease; HR, hazard ratio; CI, confidence interval. *P<0.05,**P<0.01,***P<0.001.

Furthermore, we performed subgroup analyses based on sex, HBA1c level, and CKD stages, and found that SUA was a more independent risk factor for ESRD in patients with HBA1c less than 7% and younger than 40 years of age. as shown in **Table 5**.

**Table 5:** subgroup analysis of risk factors associated to ESRD in 155 DKD patients.

Subgroup	n (%)	HR (95%CI)		P value
All patients	155 (100.00)	1.00 (1.00 ~ 1.01)	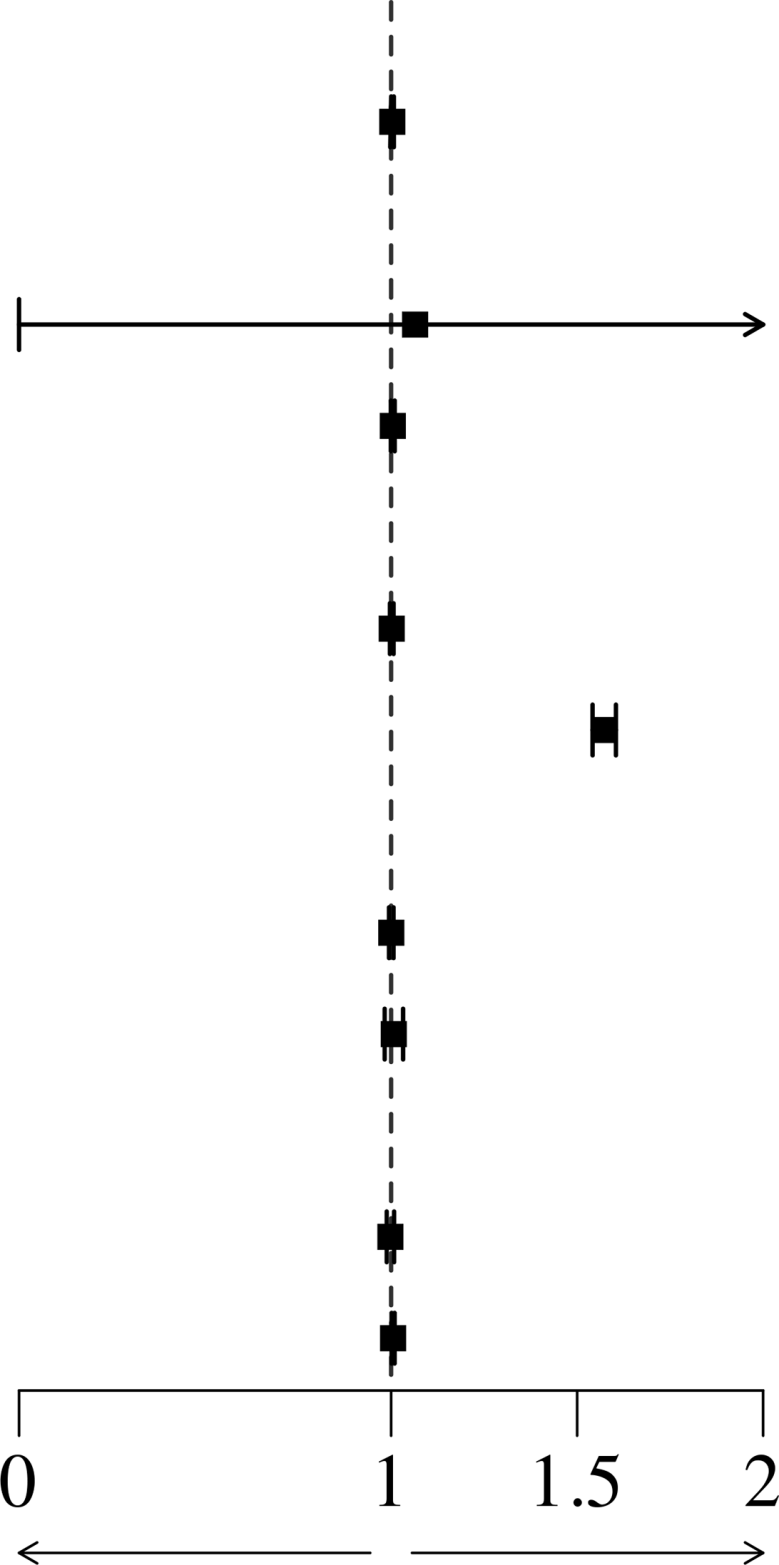	0.137
Gender				
Male	39 (25.16)	1.06 (0.00 ~ 17036340189167112192.00)		0.998
Female	116 (74.84)	1.00 (1.00 ~ 1.01)		0.082
Age				
≥ 40 year	133 (85.81)	1.00 (1.00 ~ 1.01)		0.509
< 40 year	22 (14.19)	1.57 (1.54 ~ 1.60)		<.001
CKD				
1-3	129 (83.23)	1.00 (0.99 ~ 1.01)		0.893
4-5	26 (16.77)	1.01 (0.98 - 1.03)		0.571
HbA1c				
≥ 7%	79 (50.97)	1.00 (0.99 - 1.01)		0.701
< 7%	76 (49.03)	1.01 (1.00 ~ 1.01)		0.010

### Risk prediction model based on COX

**Figure 3:**
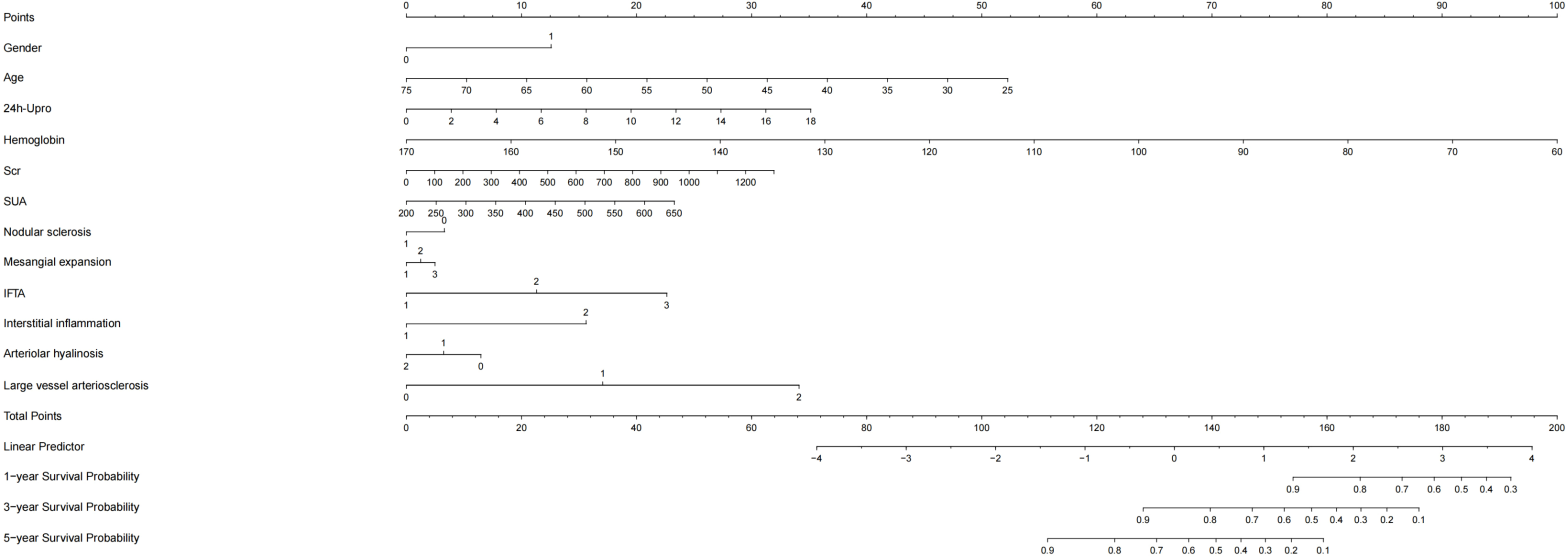
Nomogram model of kidney survival in DKD patients based on COX. 24h-Upor, 24-hour proteinuria; SUA, serum uric acid; IFTA, interstitial fibrosis and tubular atrophy; Scr, serum creatinine; DKD, Diabetic kidney disease.

Based on the Cox regression results, the VIMP method and minimum depth method were combined to select variables, and a total of 11 variables were obtained, including age, sex, 24 h Upro, haemoglobin, Scr, SUA, degree of mesangial expansion, presence of nodular sclerosis, IFTA, interstitial inflammation grade, vessel arteriosclerosis and arteriolar hyalinosis. A nomogram model is shown in **Figure 3**.

**Figure 4:**
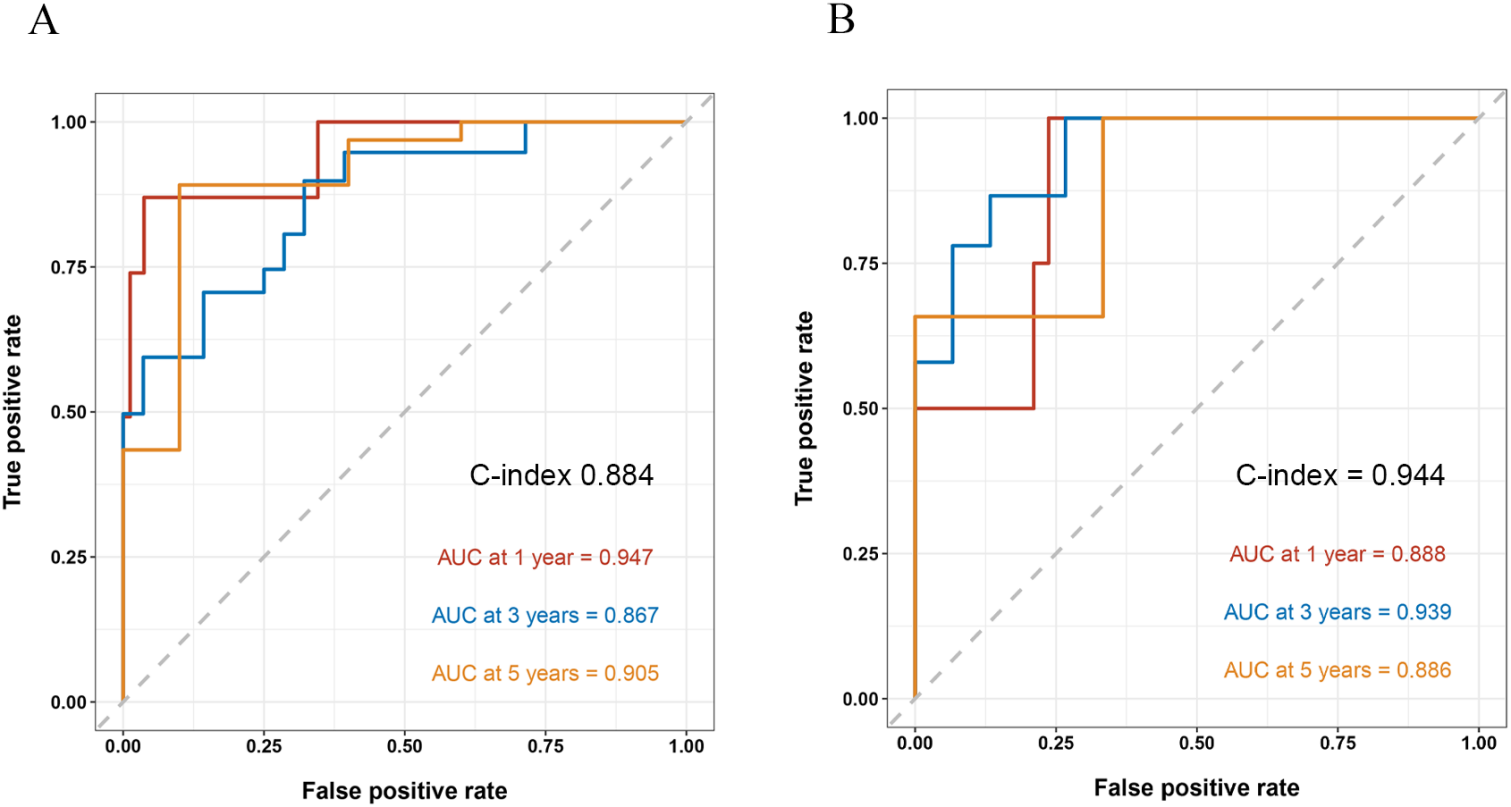
ROC curve and the AUC calculated for both the training and testing sets. AUC, area under the receiver operating curve.

By internal validation in the training set and testing sets. This model was predictive with high accuracy. Furthermore, the ROC curve was constructed, and the AUC was calculated for both the training and testing sets. The AUCs at 1, 3, and 5 years were 0.947, 0.867, and 0.905 in the training testing set, C-index=0.884 (**Figure 4A**), and 0.888, 0.939 and 0.886 in the testing set, C-index=0.944 (**Figure 4B**), indicating that the model had high discrimination.

## Discussion

Many studies have reported that SUA is an important factor for the risk and progression of DKD. Most of these studies of DKD have been conducted based on clinical diagnosis: patients with type I or type II diabetes, persistent proteinuria: albumin creatinine ratio (ACR)≥30 mg/g, eGFR<60 ml/min, kidney transplantation, haemodialysis, or peritoneal dialysis. However, it is widely known that DKD is often associated with primary glomerular diseases (IgA nephropathy, membranous nephropathy, etc.), so the clinical diagnostic criteria have great limitations, and the correlation between SUA levels and pathological manifestations cannot be evaluated. To the best of our knowledge, there was limit literature take the diagnosis of renal pathology as the admission criterion of DKD and analyse the correlation between SUA level and renal pathological diagnosis and prognosis. In our study, we found IFTA score was significantly higher in the hyperuricaemia group, and the linear correlation analysis showed that the IFTA score and the SUA level were linearly positively correlated, which consistent with Zou’s report (10). Inflammation is the main cause of tubulointerstitial injury. Many studies have reported that SUA elicits an inflammatory response characterized by the secretion of proinflammatory mediators and the recruitment of leucocytes from the bloodstream into the inflamed tissue. Through the JNK pathway, SUA can directly activate macrophages, leading to cytokine production (11). Kim et al. reported that SUA directly resulted in interleukin 1β (IL-1β) production by activating the NLRP3 inflammasome complex (12). Uric acid was also shown to induce alterations in the nitric oxide (NO) pathway and activate the renin-angiotensin system, suggesting possible links between SUA and kidney injury (13, 14).Therefore, the activation of inflammatory pathways caused by UA is an important cause of more serious tubulointerstitial injury. In addition to IFTA, we also found a higher degree of mesangial expansion in patients with hyperuricaemia. Mesangial expansion is the result of matrix deposition. Animal studies have shown that UA promotes mesangial cell activation and mesangial matrix secretion by activating NADPH/ROS/ERK1/2 signaling (15). The mesangial deposition and expansion eventually lead to nodular sclerosis which is thecharacteristic pathological change in late DKD. Therefore, these results indicted that level of SUA has greater correlation with the pathological changes of early DKD.

The prevalence of hyperuricaemia among patients with T2DM is as high as 20.68%~33.8%. The SUA level was positively correlated with an increased risk of DKD. However, current research on the association between SUA and DKD progression remains controversial. In two T1D studies, high levels of baseline SUA were found to be strongly associated with the occurrence of albuminuria during follow-up (16, 17). With every 100 μmol/L increase in SUA, the risk of macroalbuminuria increased by 2.93 times, while baseline SUA did not predict the risk of microalbuminuria or GFR loss (16). Another study with 355 T1D patients found a progressively increasing risk of rapid eGFR decline with SUA levels increasing from < 3.0 to > 6.0 mg/dL (18). THayashino et al. enrolled 2518 T2D patients who were followed up for 2 years, and the endpoints were defined as albuminuria progression. The results showed a U-shaped curve of the influence of SUA on the progression of DKD and an increased hazard for the lowest SUA quartile (19). Among 768 T2DM patients, Zhu and his colleagues reported that patients with SUA >420 μmol/L were ninefold more likely to be in a higher KDIGO risk category than those with SUA <300 μmol/L, which indicated that hyperuricaemia may be associated with a higher risk of DKD progression in individuals with T2D (20). In our study we found that patients with hyperuricaemia had more serious clinical changes including higher serum creatinine, total cholesterol, low-density lipoprotein, and blood phosphate concentrations and lower GFR and haemoglobin levels and Kaplan-Meier analysis revealed a significant difference in the cumulative renal survival among the two groups. Landmark analysis revealed that patients in the hyperuricaemia group had worse survival than those in the nonhyperuricaemia group after the first follow-up at 12 months, which indicated the influence of SUA on long-term renal prognosis. However, In 2021,Zou’s research showed a different result. No correlation was found between SUA levels and renal outcomes (10).This may be due to the fact that the patients included in the study had more severe clinical and pathological manifestations, advanced disease, and a longer follow-up period. The effect of Scr and proteinuria on prognosis in the late stage of DKD was much greater than SUA, which further confirms the significance of SUA level in the prognosis of early DKD. The influence of uric acid on renal prognosis is mainly related to the special biological characteristics of uric acid which include pro-oxidant, pro-inflammatory, nitric oxide regulation and immune system interaction (21). Renin-angiotensin system activation plays a crucial role in the progression of DKD. SUA exerts its influence on the renin-angiotensin system through mechanisms involving the stimulation of plasma renin activity, renal renin expression and activation of the intrarenal angiotensin system (22) Besides Activated ARS system will lead to increased secretion of downstream aldosterone. A recent study has found that plasma aldosterone level is closely related to SUA level in hypertensive patients and is an independent risk factor for hyperuricosis and gout (23).Aldosterone might through induce inflammation, triggering various inflammatory markers which disrupt UA metabolism (24, 25) and prompts oxidative stress, activating reactive oxygen species and transcription factors that harm the kidneys (26, 27).

Compared with that of other CKD patients, patients with DKD have a higher risk of progressing to ESRD, and many factors, such as hypertension, hypoalbuminaemia, proteinuria and higher CKD stages, affect the progression of patients with DKD (28–30). In our study, based on Cox regression results, we combined 11 clinical and pathological variables (including age, sex, 24 h Upro, haemoglobin, Scr, SUA, degree of mesangial expansion, presence of nodular sclerosis, IFTA, interstitial inflammation grade, vessel arteriosclerosis and arteriolar hyalinosis) to construct a new nomogram model with higher predictive value for DKD prognosis. The AUCs at 1, 3, and 5 years were 0.888, 0.939 and 0.886 in the testing set and 0.947, 0.867, and 0.905 in the training set, illustrating that the model had high discrimination. We also performed subgroup analyses the results showed that UA play a more important impact on patients with lower HBA1c level. This is likely because hyperglycemia can lead to increased renal perfusion, hyperfiltration, overactivation of the renin-angiotensin system (RAS). Advanced glycation end products can induce more severe oxidative stress, resulting in damage to various intrinsic renal cells such as podocytes, endothelial cells, and tubular epithelial cells, thereby leading to more significant renal impairment (31). Simultaneously, our observations indicate that hyperuricemia exhibits a greater predictive value for ESRD in the subgroup of individuals under the age of 40. Research suggests that the alteration in albuminuria associated with diabetic nephropathy demonstrates a pronounced age-dependent trend, potentially linked to the age-related increase in glomerular basement membrane permeability (32). Furthermore, factors contributing to renal function decline, including elevated blood pressure and cardiovascular events, are more prevalent with advancing age. In addition, kidney function declines faster in the elderly population, thus diluting the impact of high uric acid on kidney function. Therefore, in summary, we believe that in young patients with good blood glucose control, strict management of uric acid is more necessary to try to reduce the risk of ESRD, thus diluting the impact of high uric acid on kidney function.

Our study has various limitations. First, the patients included in our study covered ckd1-5. Due to the limited number of samples, subgroup comparisons for different CKD stages were not conducted, and the influence of Scr on the outcome could not be completely excluded. Second, the follow-up time was short, and the follow-up data of enrolled patients were incomplete. Third, there may be selection bias in our patients undergoing kidney biopsy, because only patients with more severe clinical presentations undergo biopsy, which may limit the generalizability. Therefore, future studies should be conducted to compare the clinical and prognostic differences between patients with clinically diagnosed DKD and those with biopsy-confirmed DKD. Fourth, our study is a single-centre retrospective study, which can only represent the results of this centre and lacks universality, in the future we will further conduct multi-center, prospective studies to better validate these findings.

## Conclusion

Despite these limitations of our study, we also identified the link between baseline SUA level and progression of DKD in patients with T2D, and showed that SUA is involved in the progression of DKD mainly by influencing tubulointerstitial lesions. Compared to the recent impact, baseline uric acid levels have a greater impact on the long-term prognosis of DKD. The new predictive model, which combined age, sex, 24 h Upro, haemoglobin, Scr, SUA, degree of mesangial expansion, presence of nodular sclerosis, IFTA, interstitial inflammation grade, vessel arteriosclerosis and arteriolar hyalinosis, can better help clinicians evaluate the prognosis of patients with diabetic nephropathy patients, and guide further treatment.

## Data Availability

The original contributions presented in the study are included in the article/supplementary material. Further inquiries can be directed to the corresponding author.
